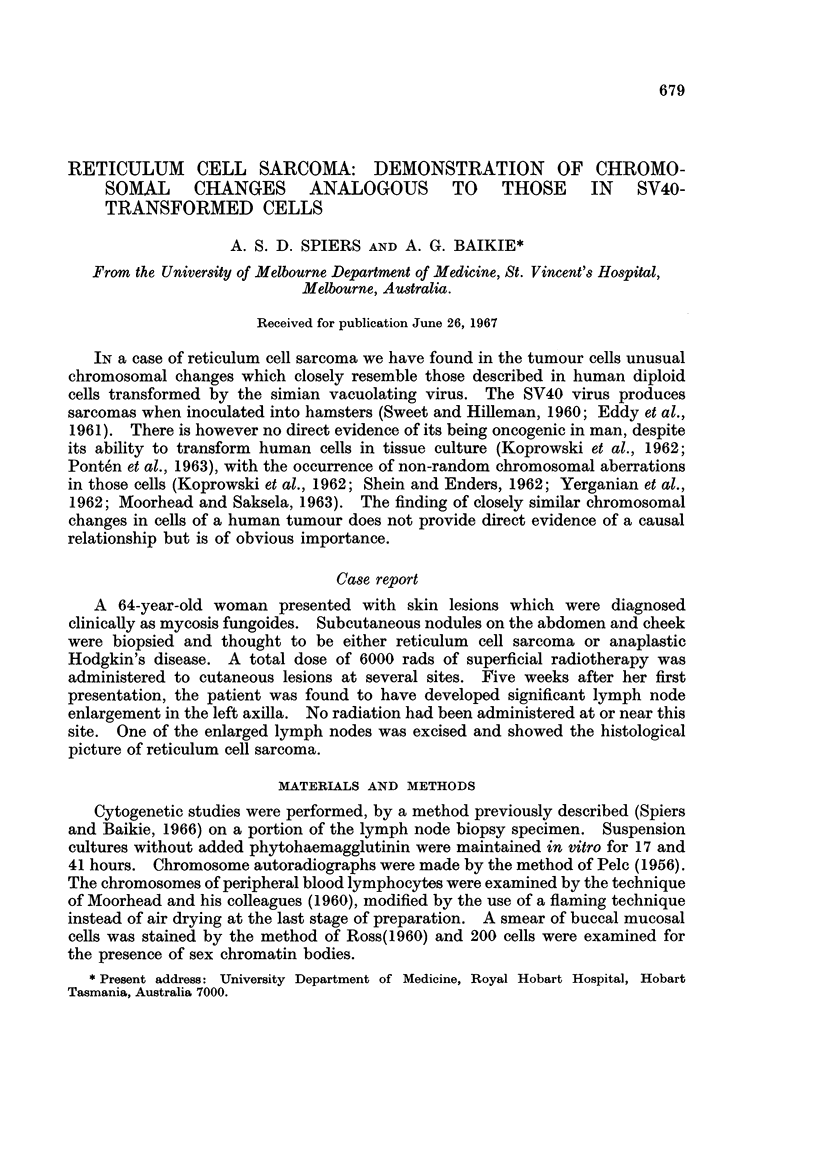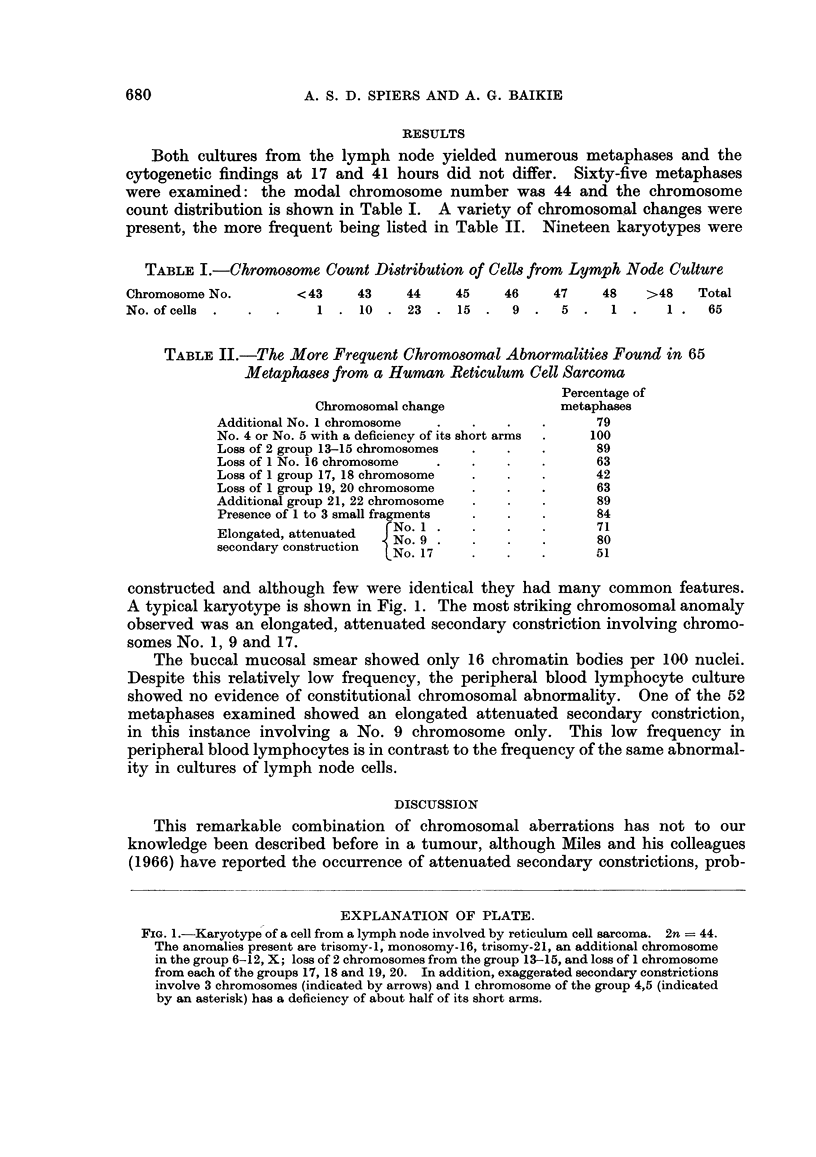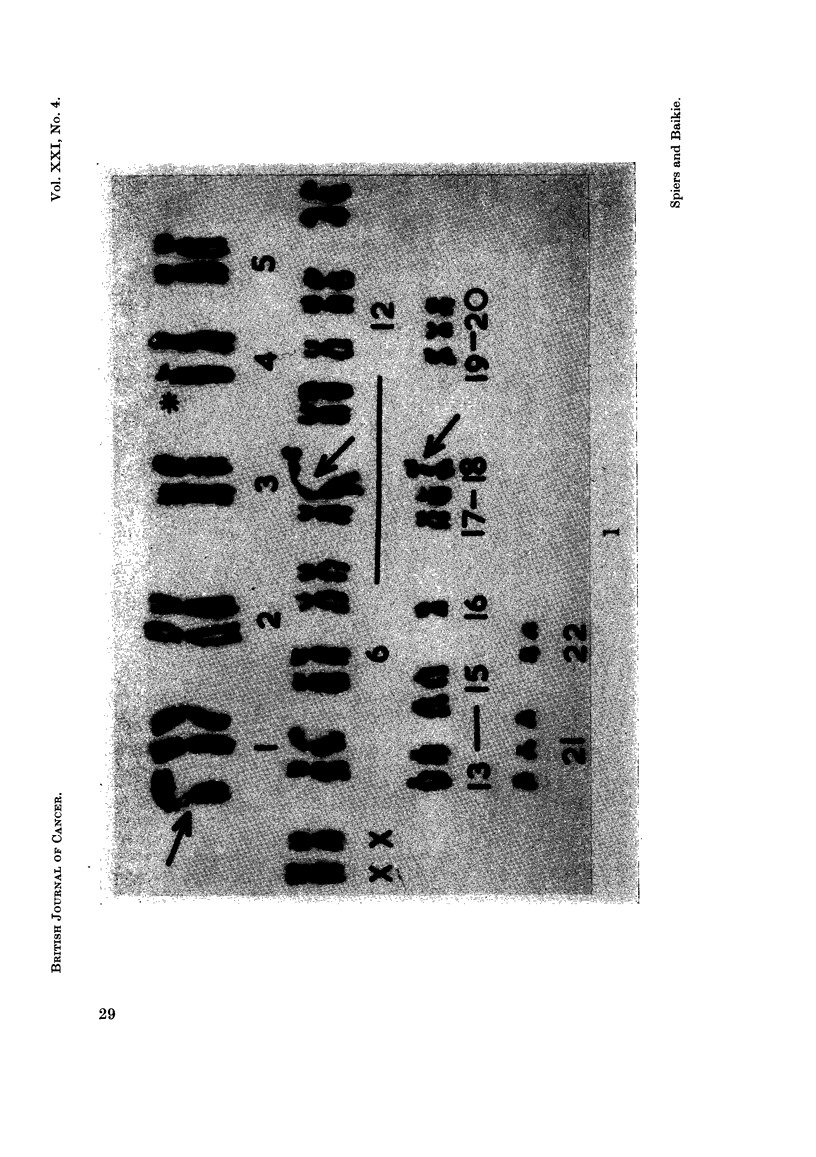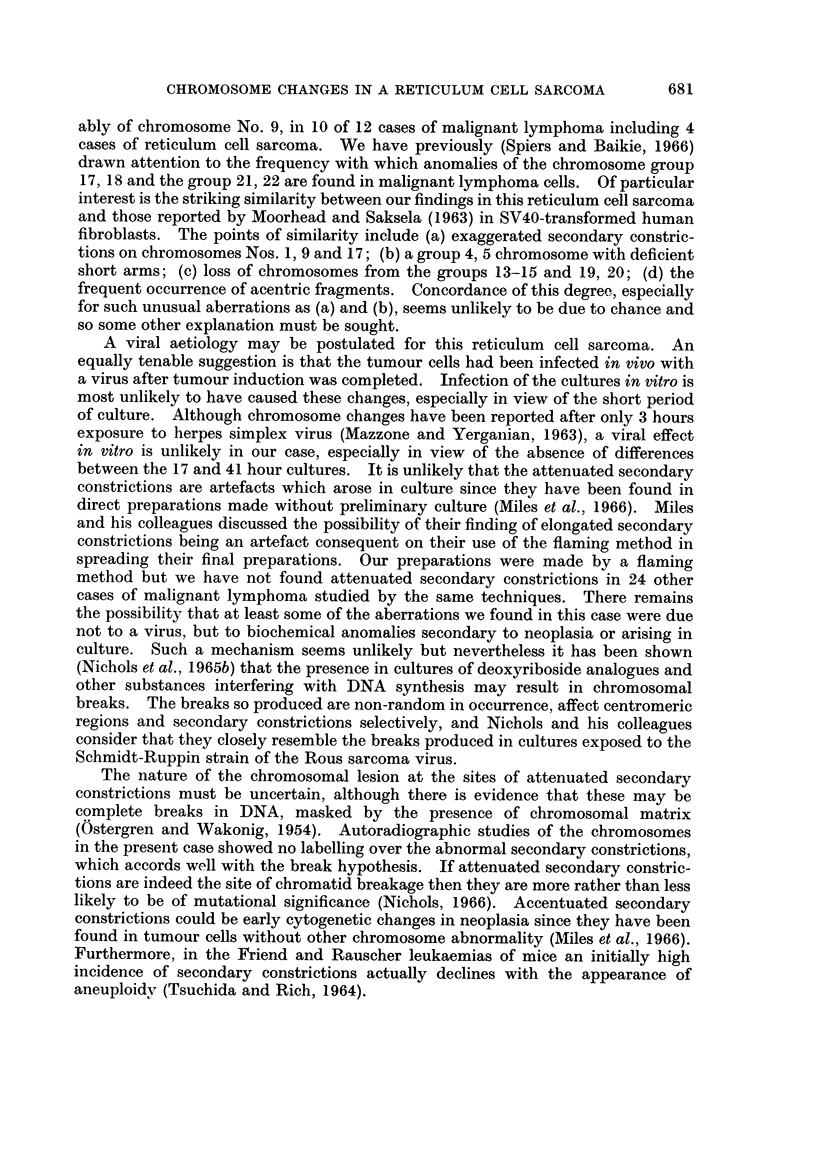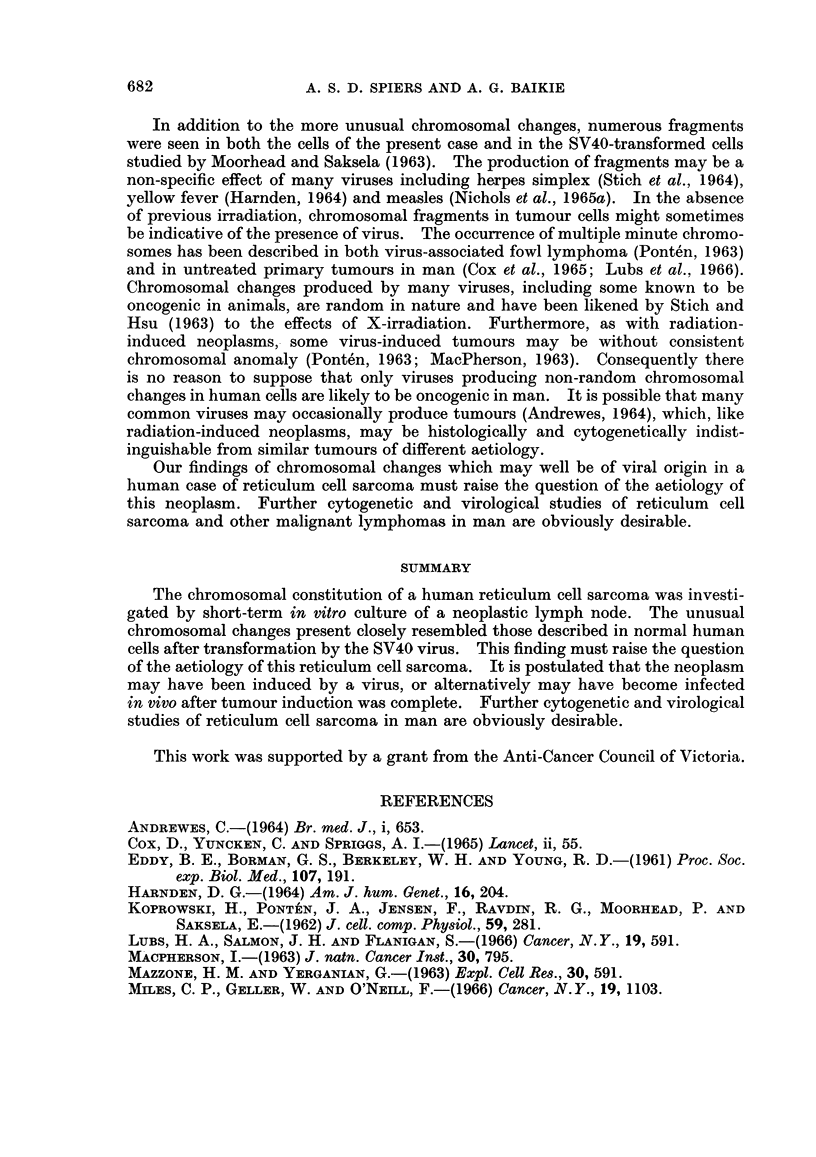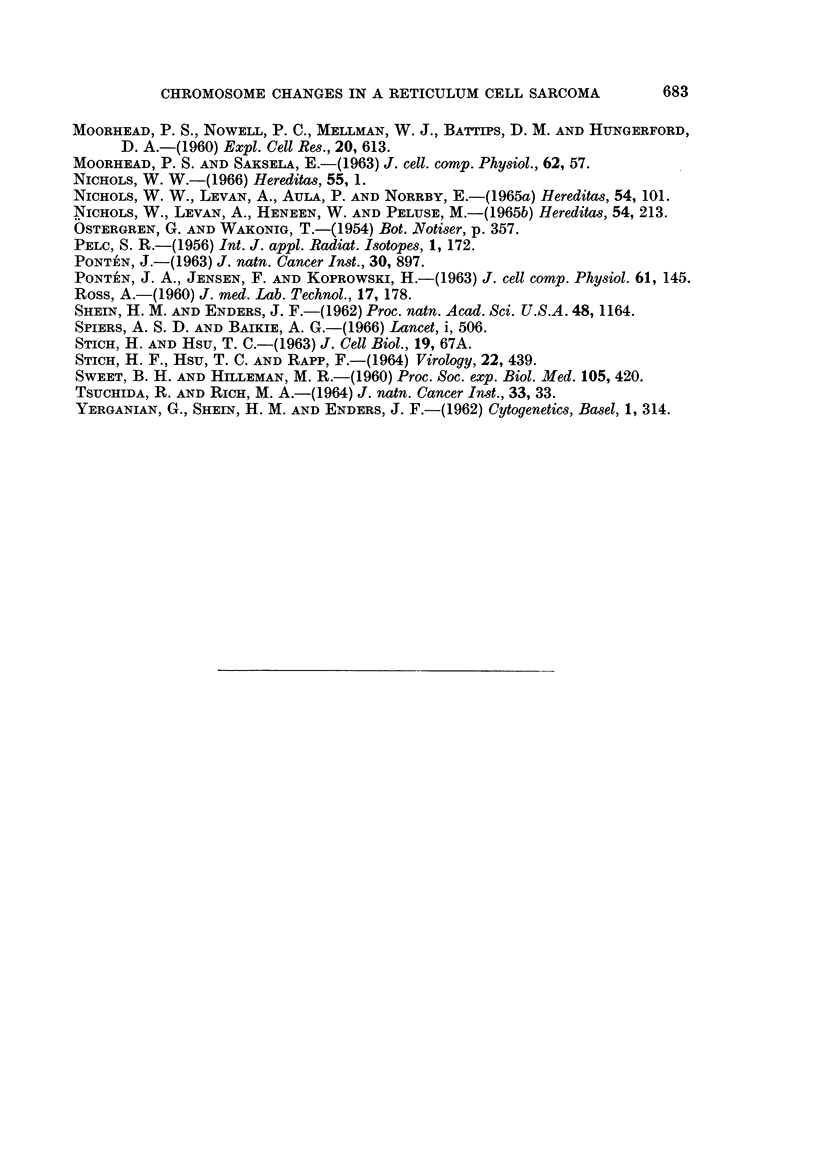# Reticulum cell sarcoma: demonstration of chromosomal changes analogous to those in SV40-transformed cells.

**DOI:** 10.1038/bjc.1967.80

**Published:** 1967-12

**Authors:** A. S. Spiers, A. G. Baikie

## Abstract

**Images:**


### RETICULUM CELL SARCOMA: DEMONSTRATION OF CHROMOSOMAL CHANGES ANALOGOUS TO THOSE IN SV40-TRANSFORMED CELLS

A. S. D. SPIERS AND A. G. BAIKIE*

*From the University of Melbourne Department of Medicine, St. Vincent's Hospital, Melbourne, Australia.*

Received for publication June 26, 1967

IN a case of reticulum cell sarcoma we have found in the tumour cells unusual chromosomal changes which closely resemble those described in human diploid cells transformed by the simian vacuolating virus. The SV40 virus produces sarcomas when inoculated into hamsters (Sweet and Hilleman, 1960; Eddy *et al.*, 1961). There is however no direct evidence of its being oncogenic in man, despite its ability to transform human cells in tissue culture (Koprowski *et al.*, 1962; Pontén *et al.*, 1963), with the occurrence of non-random chromosomal aberrations in those cells (Koprowski *et al.*, 1962; Shein and Enders, 1962; Yerganian *et al.*, 1962; Moorhead and Saksela, 1963). The finding of closely similar chromosomal changes in cells of a human tumour does not provide direct evidence of a causal relationship but is of obvious importance.

### *Case report*

A 64-year-old woman presented with skin lesions which were diagnosed clinically as mycosis fungoides. Subcutaneous nodules on the abdomen and cheek were biopsied and thought to be either reticulum cell sarcoma or anaplastic Hodgkin's disease. A total dose of 6000 rads of superficial radiotherapy was administered to cutaneous lesions at several sites. Five weeks after her first presentation, the patient was found to have developed significant lymph node enlargement in the left axilla. No radiation had been administered at or near this site. One of the enlarged lymph nodes was excised and showed the histological picture of reticulum cell sarcoma.

### MATERIALS AND METHODS

Cytogenetic studies were performed, by a method previously described (Spiers and Baikie, 1966) on a portion of the lymph node biopsy specimen. Suspension cultures without added phytohaemagglutinin were maintained *in vitro* for 17 and 41 hours. Chromosome autoradiographs were made by the method of Pelc (1956). The chromosomes of peripheral blood lymphocytes were examined by the technique of Moorhead and his colleagues (1960), modified by the use of a flaming technique instead of air drying at the last stage of preparation. A smear of buccal mucosal cells was stained by the method of Ross(1960) and 200 cells were examined for the presence of sex chromatin bodies.

* Present address: University Department of Medicine, Royal Hobart Hospital, Hobart Tasmania, Australia 7000.

### RESULTS

Both cultures from the lymph node yielded numerous metaphases and the cytogenetic findings at 17 and 41 hours did not differ. Sixty-five metaphases were examined: the modal chromosome number was 44 and the chromosome count distribution is shown in Table I. A variety of chromosomal changes were present, the more frequent being listed in Table II. Nineteen karyotypes were constructed and although few were identical they had many common features. A typical karyotype is shown in Fig. 1. The most striking chromosomal anomaly observed was an elongated, attenuated secondary constriction involving chromosomes No. 1, 9 and 17.

TABLE I.—*Chromosome Count Distribution of Cells from Lymph Node Culture*

| Chromosome No. | <43 | 43 | 44 | 45 | 46 | 47 | 48 | >48 | Total |
|---|---|---|---|---|---|---|---|---|---|
| No. of cells | 1 | 10 | 23 | 15 | 9 | 5 | 1 | 1 | 65 |

TABLE II.—*The More Frequent Chromosomal Abnormalities Found in 65 Metaphases from a Human Reticulum Cell Sarcoma*

| Chromosomal change | | Percentage of metaphases |
|---|---|---|
| Additional No. 1 chromosome | | 79 |
| No. 4 or No. 5 with a deficiency of its short arms | | 100 |
| Loss of 2 group 13–15 chromosomes | | 89 |
| Loss of 1 No. 16 chromosome | | 63 |
| Loss of 1 group 17, 18 chromosome | | 42 |
| Loss of 1 group 19, 20 chromosome | | 63 |
| Additional group 21, 22 chromosome | | 89 |
| Presence of 1 to 3 small fragments | | 84 |
| Elongated, attenuated secondary construction | No. 1 | 71 |
| | No. 9 | 80 |
| | No. 17 | 51 |

The buccal mucosal smear showed only 16 chromatin bodies per 100 nuclei. Despite this relatively low frequency, the peripheral blood lymphocyte culture showed no evidence of constitutional chromosomal abnormality. One of the 52 metaphases examined showed an elongated attenuated secondary constriction, in this instance involving a No. 9 chromosome only. This low frequency in peripheral blood lymphocytes is in contrast to the frequency of the same abnormality in cultures of lymph node cells.

### DISCUSSION

This remarkable combination of chromosomal aberrations has not to our knowledge been described before in a tumour, although Miles and his colleagues (1966) have reported the occurrence of attenuated secondary constrictions, prob-

---

EXPLANATION OF PLATE.

FIG. 1.—Karyotype of a cell from a lymph node involved by reticulum cell sarcoma. $2n = 44$. The anomalies present are trisomy-1, monosomy-16, trisomy-21, an additional chromosome in the group 6–12, X; loss of 2 chromosomes from the group 13–15, and loss of 1 chromosome from each of the groups 17, 18 and 19, 20. In addition, exaggerated secondary constrictions involve 3 chromosomes (indicated by arrows) and 1 chromosome of the group 4,5 (indicated by an asterisk) has a deficiency of about half of its short arms.

1

Spiers and Baikie.

ably of chromosome No. 9, in 10 of 12 cases of malignant lymphoma including 4 cases of reticulum cell sarcoma. We have previously (Spiers and Baikie, 1966) drawn attention to the frequency with which anomalies of the chromosome group 17, 18 and the group 21, 22 are found in malignant lymphoma cells. Of particular interest is the striking similarity between our findings in this reticulum cell sarcoma and those reported by Moorhead and Saksela (1963) in SV40-transformed human fibroblasts. The points of similarity include (a) exaggerated secondary constrictions on chromosomes Nos. 1, 9 and 17; (b) a group 4, 5 chromosome with deficient short arms; (c) loss of chromosomes from the groups 13–15 and 19, 20; (d) the frequent occurrence of acentric fragments. Concordance of this degree, especially for such unusual aberrations as (a) and (b), seems unlikely to be due to chance and so some other explanation must be sought.

A viral aetiology may be postulated for this reticulum cell sarcoma. An equally tenable suggestion is that the tumour cells had been infected *in vivo* with a virus after tumour induction was completed. Infection of the cultures *in vitro* is most unlikely to have caused these changes, especially in view of the short period of culture. Although chromosome changes have been reported after only 3 hours exposure to herpes simplex virus (Mazzone and Yerganian, 1963), a viral effect *in vitro* is unlikely in our case, especially in view of the absence of differences between the 17 and 41 hour cultures. It is unlikely that the attenuated secondary constrictions are artefacts which arose in culture since they have been found in direct preparations made without preliminary culture (Miles *et al.*, 1966). Miles and his colleagues discussed the possibility of their finding of elongated secondary constrictions being an artefact consequent on their use of the flaming method in spreading their final preparations. Our preparations were made by a flaming method but we have not found attenuated secondary constrictions in 24 other cases of malignant lymphoma studied by the same techniques. There remains the possibility that at least some of the aberrations we found in this case were due not to a virus, but to biochemical anomalies secondary to neoplasia or arising in culture. Such a mechanism seems unlikely but nevertheless it has been shown (Nichols *et al.*, 1965*b*) that the presence in cultures of deoxyriboside analogues and other substances interfering with DNA synthesis may result in chromosomal breaks. The breaks so produced are non-random in occurrence, affect centromeric regions and secondary constrictions selectively, and Nichols and his colleagues consider that they closely resemble the breaks produced in cultures exposed to the Schmidt-Ruppin strain of the Rous sarcoma virus.

The nature of the chromosomal lesion at the sites of attenuated secondary constrictions must be uncertain, although there is evidence that these may be complete breaks in DNA, masked by the presence of chromosomal matrix (Östergren and Wakonig, 1954). Autoradiographic studies of the chromosomes in the present case showed no labelling over the abnormal secondary constrictions, which accords well with the break hypothesis. If attenuated secondary constrictions are indeed the site of chromatid breakage then they are more rather than less likely to be of mutational significance (Nichols, 1966). Accentuated secondary constrictions could be early cytogenetic changes in neoplasia since they have been found in tumour cells without other chromosome abnormality (Miles *et al.*, 1966). Furthermore, in the Friend and Rauscher leukaemias of mice an initially high incidence of secondary constrictions actually declines with the appearance of aneuploidy (Tsuchida and Rich, 1964).

In addition to the more unusual chromosomal changes, numerous fragments were seen in both the cells of the present case and in the SV40-transformed cells studied by Moorhead and Saksela (1963). The production of fragments may be a non-specific effect of many viruses including herpes simplex (Stich *et al.*, 1964), yellow fever (Harnden, 1964) and measles (Nichols *et al.*, 1965*a*). In the absence of previous irradiation, chromosomal fragments in tumour cells might sometimes be indicative of the presence of virus. The occurrence of multiple minute chromosomes has been described in both virus-associated fowl lymphoma (Pontén, 1963) and in untreated primary tumours in man (Cox *et al.*, 1965; Lubs *et al.*, 1966). Chromosomal changes produced by many viruses, including some known to be oncogenic in animals, are random in nature and have been likened by Stich and Hsu (1963) to the effects of X-irradiation. Furthermore, as with radiation-induced neoplasms, some virus-induced tumours may be without consistent chromosomal anomaly (Pontén, 1963; MacPherson, 1963). Consequently there is no reason to suppose that only viruses producing non-random chromosomal changes in human cells are likely to be oncogenic in man. It is possible that many common viruses may occasionally produce tumours (Andrewes, 1964), which, like radiation-induced neoplasms, may be histologically and cytogenetically indistinguishable from similar tumours of different aetiology.

Our findings of chromosomal changes which may well be of viral origin in a human case of reticulum cell sarcoma must raise the question of the aetiology of this neoplasm. Further cytogenetic and virological studies of reticulum cell sarcoma and other malignant lymphomas in man are obviously desirable.

### SUMMARY

The chromosomal constitution of a human reticulum cell sarcoma was investigated by short-term *in vitro* culture of a neoplastic lymph node. The unusual chromosomal changes present closely resembled those described in normal human cells after transformation by the SV40 virus. This finding must raise the question of the aetiology of this reticulum cell sarcoma. It is postulated that the neoplasm may have been induced by a virus, or alternatively may have become infected *in vivo* after tumour induction was complete. Further cytogenetic and virological studies of reticulum cell sarcoma in man are obviously desirable.

This work was supported by a grant from the Anti-Cancer Council of Victoria.

### REFERENCES

ANDREWES, C.—(1964) *Br. med. J.*, i, 653.
COX, D., YUNCKEN, C. AND SPRIGGS, A. I.—(1965) *Lancet*, ii, 55.
EDDY, B. E., BORMAN, G. S., BERKELEY, W. H. AND YOUNG, R. D.—(1961) *Proc. Soc. exp. Biol. Med.*, **107**, 191.
HARNDEN, D. G.—(1964) *Am. J. hum. Genet.*, **16**, 204.
KOPROWSKI, H., PONTÉN, J. A., JENSEN, F., RAVDIN, R. G., MOORHEAD, P. AND SAKSELA, E.—(1962) *J. cell. comp. Physiol.*, **59**, 281.
LUBS, H. A., SALMON, J. H. AND FLANIGAN, S.—(1966) *Cancer, N.Y.*, **19**, 591.
MACPHERSON, I.—(1963) *J. natn. Cancer Inst.*, **30**, 795.
MAZZONE, H. M. AND YERGANIAN, G.—(1963) *Expl. Cell Res.*, **30**, 591.
MILES, C. P., GELLER, W. AND O'NEILL, F.—(1966) *Cancer, N.Y.*, **19**, 1103.

MOORHEAD, P. S., NOWELL, P. C., MELLMAN, W. J., BATTIPS, D. M. AND HUNGERFORD, D. A.—(1960) *Expl. Cell Res.*, **20**, 613.
MOORHEAD, P. S. AND SAKSELA, E.—(1963) *J. cell. comp. Physiol.*, **62**, 57.
NICHOLS, W. W.—(1966) *Hereditas*, **55**, 1.
NICHOLS, W. W., LEVAN, A., AULA, P. AND NORRBY, E.—(1965*a*) *Hereditas*, **54**, 101.
NICHOLS, W., LEVAN, A., HENEEN, W. AND PELUSE, M.—(1965*b*) *Hereditas*, **54**, 213.
ÖSTERGREN, G. AND WAKONIG, T.—(1954) *Bot. Notiser*, p. 357.
PELC, S. R.—(1956) *Int. J. appl. Radiat. Isotopes*, **1**, 172.
PONTÉN, J.—(1963) *J. natn. Cancer Inst.*, **30**, 897.
PONTÉN, J. A., JENSEN, F. AND KOPROWSKI, H.—(1963) *J. cell comp. Physiol.* **61**, 145.
ROSS, A.—(1960) *J. med. Lab. Technol.*, **17**, 178.
SHEIN, H. M. AND ENDERS, J. F.—(1962) *Proc. natn. Acad. Sci. U.S.A.* **48**, 1164.
SPIERS, A. S. D. AND BAIKIE, A. G.—(1966) *Lancet*, i, 506.
STICH, H. AND HSU, T. C.—(1963) *J. Cell Biol.*, **19**, 67A.
STICH, H. F., HSU, T. C. AND RAPP, F.—(1964) *Virology*, **22**, 439.
SWEET, B. H. AND HILLEMAN, M. R.—(1960) *Proc. Soc. exp. Biol. Med.* **105**, 420.
TSUCHIDA, R. AND RICH, M. A.—(1964) *J. natn. Cancer Inst.*, **33**, 33.
YERGANIAN, G., SHEIN, H. M. AND ENDERS, J. F.—(1962) *Cytogenetics, Basel*, **1**, 314.